# Treatment of insomnia based on the mechanism of pathophysiology by acupuncture combined with herbal medicine: A review

**DOI:** 10.1097/MD.0000000000033213

**Published:** 2023-03-17

**Authors:** Jie Wang, Haishen Zhao, Kejun Shi, Manya Wang

**Affiliations:** a Department of Pain, Datong Hospital of Traditional Chinese Medicine, Shanxi Province, Datong, China; b Department of Rehabilitation, Luchaogang Community Health Service Center, Pudong New District, Shanghai, China.

**Keywords:** acupuncture, Chinese herbal formula, Chinese medicine, genetics, heart-brain axis, herbal medicine, herbal medicine, insomnia, neurobiology, sleep mechanism

## Abstract

Insomnia is a sleep disorder which severely affects patients mood, quality of life and social functioning, serves as a trigger or risk factor to a variety of diseases such as depression, cardiovascular and cerebrovascular diseases, obesity and diabetes, and even increases the risk of suicide, and has become an increasingly widespread concern worldwide. Considerable research on insomnia has been conducted in modern medicine in recent years and encouraging results have been achieved in the fields of genetics and neurobiology. Unfortunately, however, the pathogenesis of insomnia remains elusive to modern medicine, and pharmacological treatment of insomnia has been regarded as conventional. However, in the course of treatment, pharmacological treatment itself is increasingly being questioned due to potential dependence and drug resistance and is now being replaced by cognitive behavior therapy as the first-line treatment. As an important component of complementary and alternative medicine, traditional Chinese medicine, especially non-pharmacological treatment methods such as acupuncture, is gaining increasing attention worldwide. In this article, we discuss the combination of traditional Chinese medicine, acupuncture, and medicine to treat insomnia based on neurobiology in the context of modern medicine.

## 1. Introduction

### 1.1. Modern medicine perception of insomnia

Insomnia is used to describe the experience of difficulty in sleeping. When people have sufficient time and a subjective desire to fall asleep but have difficulty falling asleep or have poor sleep quality and wake up easily, people who suffer from sleep disturbance have negative emotions and affect their normal work and life, which they describe as insomnia.^[1]^ Insomnia can be acute, chronic, or intermittent, and is considered the most common sleep problem in related investigations^[1]^ and can be associated with other diseases and sleep disorders. In the latest diagnostic etiology, it is proposed that insomnia is not congenitally secondary to other diseases, but co morbid with other diseases, especially with obstructive sleep apnea syndrome and restless legs syndrome are highly co morbid.^[2]^ However, this does not mean that insomnia is not secondary to other diseases, and when this happens, other diseases are successfully treated, and insomnia symptoms disappear.

In the medical literature, insomnia is often referred to simply as “insomnia, but in this article, it is always referred to as “insomnia to distinguish it from other disorders that cause insomnia symptoms. Insomnia can be chronic (onset > 3 months) or acute (onset ≤ 3 months), and in the literature it has been classified as “primary insomnia, “secondary insomnia, and other different types of chronic insomnia (e.g., Psychophysiological insomnia), but this perception is now outdated and anachronistic, and the academic community has replaced these old, outdated names with the more widely accepted “insomnia.”^[3]^ Although insomnia can occur in healthy individuals and may even be common, it can still be diagnosed as a disorder.

### 1.2. Traditional Chinese medicine perception of insomnia

In traditional Chinese medicine (TCM) literature reviews, there is an extensive and profound understanding of insomnia. There are different names such as “sleeplessness,” “lack of sleep,” “no night” and “insomnia.” The development, etiology, and diagnosis of insomnia are mentioned in many places in Yellow Emperor’s Classic of Internal Medicine, a pre-Qin text. For example, in “Ling Shu–Ying Health Hui”^[4]^: “Ying Qi is weakened and less, and Wei Qi is internally felled, so the day is not refined and the night is not close”; “Ling Shu–Evil Guest”^[4]^: “If Yang Qi is strong, Yang stilts are trapped, and the eyes are not close because of Yin deficiency.” The Suwen–the theory of inverse regulation^[5]^ said “the stomach is not harmonious, then restlessness.” This reflects the simple perception of insomnia of ancient times. In later medical texts, there were 2 relatively different views on insomnia. One of them is that “the loss of nourishment of the heart and mind” is the fundamental reason for the development of insomnia, that is, the cloud of “Continuing the Case of Famous Physicians”: “When a person sleeps peacefully, the mind returns to the heart and the 5 viscera each rest in its place and sleep peacefully”; and “The Book of Jing Yue Quan Shu—Insomnia.” Jing Yue Quan Shu-sleeplessness said: “The mind is safe then sleep; the mind is restless with sleeplessness.” It is important to note that the “heart” in Chinese medicine does not exactly correspond to the heart organ in modern anatomy, nor can it be simply and coarsely equated. In Chinese medicine, the heart is considered to be the “sovereign” that commands the whole organism and is involved in the pathogenesis and treatment of insomnia as the “place of consciousness,” and is considered to be the “seat of the gods” (spirit) or mind. The “mind” is also the seat of the body as a “sovereign” and is involved in the pathogenesis and treatment of insomnia. The “heart” is also a central point involved in the etiology and pathogenesis of anxiety and depression. Addressing the imbalance associated with the “heart” is a key strategy in the treatment of insomnia in TCM. There is scientific evidence that acupuncture can cause physiological changes in the heart,^[7]^ and from a modern medical perspective, these changes are related to the pathophysiology of insomnia. Although this traditional concept seems inconsistent with the modern physiological understanding when first examined, it does make sense in terms of the function of the vagus nervous system. The vagus nerve (10th cerebral nerve) is primarily the afferent nerve that informs the visceral experience of the brain. The ancient Chinese view that consciousness and thought are attributed to the “heart” is of research interest and value.

Another view is that the “brain” is the essence of insomnia, and the “brain” is considered as the “House of the Gods” in Chinese medicine,^[8]^ which corresponds to the “brain in modern medicine. In Chinese medicine, “brain is considered to be the “house of the vital spirit,”^[8]^ which corresponds to “brain in modern medicine. In Chinese medicine, the brain is considered to be the master of thinking, as stated in the Preparedness and Emergency Thousand Gold Essentials: “The head is the head of the body and the method of the human spirit, and Wang Ang of the Qing dynasty believed in the Materia Medica that “the memory of human beings is all in the brain.”^[8]^ Wang Qingren, in Materia Medica, argued that “the brain is the highest level of the nervous system, not the heart, but the brain’.^[9]^ Although Chinese medicine has a certain understanding of the physiology and pathology of the brain, it is still guided in clinical practice by the theory of Tibetan elephants. In Medical Zhongzheng Ginseng Xilu, it is stated that “the brain is the yuan shen...... brain is disturbed...... shen shen is disturbed then insomnia,” and it is believed that “the brain is disturbed “The loss of nourishment in the brain will lead to the state of “Yin deficiency and Yang hyperactivity” in Chinese medicine, which affects the mental activity and nervous system of people, thus causing insomnia.

Over a long period of time, Chinese medicine has developed a rich and unique understanding of insomnia, which is to some extent common to modern medical research, but it should be noted that this understanding is based on the Tibetan theory of Chinese medicine and the fundamental principle of diagnosis and treatment in clinical practice. Owing to the complexity and uniqueness of Chinese medicine, it also presents a unique perspective that is different from that of modern medicine. In this study, we explored the possibility of combining TCM with cognitive behavioral therapy to treat insomnia based on modern medical research on the neurobiological mechanisms of insomnia.

### 1.3. The mechanism of sleep and wakefulness (Fig. 1)

To understand insomnia, we first need to understand the basis of sleep; sleep and wakefulness are normal physiological states of the brain, and 2 processes, sleep-wake homeostasis and circadian rhythm, govern the sleep-wake cycle. Sleep can be consolidated when the 2 can be combined.^[3]^ An interesting new idea is that the sleep-wake cycle embodies an organizing principle during which each process is independent, rather than assuming that sleep has a single function.^[10]^ However, this does not deny that neuronal plasticity also occurs during wakefulness. Sleep and wakefulness may occur simultaneously in a nonintegrated manner in different neuronal populations of the brain, and the states of wakefulness and sleep have traditionally been considered to be strictly separated in time, with the brain either in a sleep or a wakeful state. The assumption of a strict sequence of sleep-wake and a whole-brain or no-brain state has been continuously challenged in the last decade,^[11]^ with studies proposing that sleep and even may be a fundamental property of small local neural networks. Thus, individual cerebral cortices may display sleep-like states that occur somewhat independent of other cortical sleep-like states. Intracerebral recordings in rodents and humans demonstrated the presence of both sleep and wake-like neuronal activity.^[12]^ However, it should be noted that the concept of “local arousal islands” to explain the subjective arousal experience of sleep may be too simplistic.

**Figure 1.**
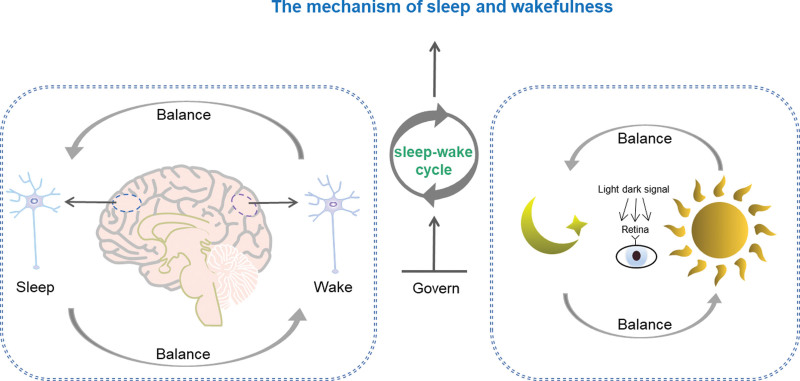
The mechanism of sleep and wakefulness.

Nevertheless, the concept of simultaneous arousal and sleep now seems to be worth investigating in depth, based on data-driven approaches showing that even patients with insomnia have more typical light sleep electroencephalogram (EEG) features than controls during deep sleep.^[13]^

### 1.4. Epidemiological studies

According to recent epidemiological studies, the overall prevalence of insomnia in China is 15%.^[14]^ Insomnia appears to be the second most common psychiatric disorder, with a 12-month prevalence between the most common co morbid anxiety disorder and major depressive disorder.^[15]^ It may be surprising that insomnia is the second most common neuropsychiatric disorder; however, the prevalence of insomnia has been increasing over the past decades, and this increased prevalence is supported by other longitudinal studies.^[16]^

Recent studies have identified females and older age groups as major determinants of insomnia prevalence.^[17]^ Although the mechanisms behind this phenomenon are not fully understood, autopsies on humans have shown gender differences in the brain structures involved in circadian rhythms and sleep regulation.^[18]^ Furthermore, animal experiments have shown that homozygous structures in females respond much more strongly to sex steroid fluctuations than those in males.^[19]^ The phase of the circadian rhythm of estradiol varies throughout the menstrual cycle, and sleep disturbances are most severe in the mid-luteal phase of the menstrual cycle, when ovarian steroid levels begin to decline and women sleep later than men, which may increase the risk of insomnia.^[19]^ Notably, female patients with major depressive disorder sleep relatively late because of their higher circadian estradiol rhythms,^[20]^ which may lead them to develop insomnia. The increased sensitivity of women’s sleep architecture and circadian rhythm regulation to sex hormone fluctuations may also contribute to the increased risk of insomnia during pregnancy and menopause.

Paradoxically, however, the higher prevalence of subjective insomnia complaints in women is not reflected in objective classical polysomnographic recordings, and it is important to note that the diagnosis of insomnia is strictly based on subjective insomnia complaints, with objective sleep criteria playing a relatively minor role.^[2]^ Conversely, objective measures suggest that women have better sleep quality than men, at least in humans. This cross-gender difference in the subjective and objective indicators of human sleep quality is only 1 notable example of our limited understanding of the neural correlates of the subjective experience of insomnia.

Epidemiological studies have shown that chronic sleep disorders, including insomnia, significantly increase with age. Frequent nocturnal awakenings were the most common age-related sleep disorder symptoms, followed by difficulty falling asleep and early awakenings.

### 1.5. Insomnia: A disorder of sleep homeostasis and regulation?

Brain circuits are primarily involved in the homeostatic components of circadian rhythm and sleep regulation. Although deviations in circadian rhythm and in vivo homeostatic regulation are likely to affect sleep quality, there is little evidence that insomnia is caused by circadian rhythm and in vivo homeostatic dysregulation.^[21]^ Regarding circadian rhythms of sleep regulation, only a few complaints of patients with insomnia are sleep complaints caused by attempts to start sleep at an inappropriate circadian phase.^[22]^ Similarly, insomnia does not appear to be caused primarily by a deficiency in the functioning of the in vivo homeostatic component of sleep regulation, and homeostatic studies have assessed how sleep deprivation alters slow wave activity in the EEG during the subsequent resumption of sleep.

Slow wave activity (SWA), which is considered a measure to assess cumulative sleep stress, increased during the baseline night in both patients with insomnia and controls, but to a lesser extent. Although some studies have shown an in vivo homeostasis deficit in insomnia,^[23]^ other studies have not confirmed this.^[24]^ All conclusions were based on studies and analyses that did not apply strict deprivation protocols regarding in vivo homeostatic sleep regulation.^[25]^ This means that insomnia may or may not have altered SWA; therefore, this is not sufficient to draw any conclusions about the lack of homeostasis in vivo.

Although adenosine has been considered a molecule that plays a key role in sleep homeostasis, with functional genetic variants in its regulation altering the duration and intensity of SWA,^[26]^ recent Genome-Wide Association Studies (GWAS) have not shown major variants in genes involved in adenosine regulation.

### 1.6. Insomnia, anxiety and depression.

According to previous studies, insomnia usually coexists with multiple neuropsychiatric disorders, with a prevalence of 80% to 90% co-morbidity with depression and anxiety.^[27]^ It should be repeatedly stated that in the past, insomnia was conceptualized as a “secondary disorder,” however, it has been demonstrated that insomnia has an independent trajectory. Insomnia does not resolve with treatment or improvement in psychiatric disorders.^[28]^ At the same time, there is a bidirectional relationship between insomnia and multiple psychiatric disorders, and insomnia may exacerbate symptoms of co morbid psychiatric disorders, impede the treatment of comorbid psychiatric disorders, and lead to an increased risk of relapse.^[3]^ A Meta-analysis of 34 cohort studies involving 150,000 participants found that the presence of insomnia doubled the relative risk of developing depression.^[29]^ A noteworthy finding was that insomnia was positively associated with an increased risk of suicidal ideation.^[30]^ Studies of patients have shown a degree of association between sleep problems and depression, and in a study of 5481 hospitalized patients, it was found that more than half of the 3108 patients whose depression was in remission at discharge still had a substantial degree of sleep disturbance^[31]^

The binary effects hypothesis suggests that common neural substrates may underlie insomnia, anxiety, and depression,^[32]^ that is, common neural substrates disrupt sleep and mental health. In depression, co-expression of the insomnia phenotype is common, and the negative effects of insomnia and emotional stress are reinforced in both directions. On the 1 hand, individuals who are very sensitive to stress are prone to insomnia^[33]^; on the other hand, preexisting insomnia puts individuals at an elevated risk of developing posttraumatic stress disorder (PTSD) when exposed to traumatic events.

The “monoamine hypothesis” proposes a role for norepinephrine in depression, with studies and findings including norepinephrine deficiency, prolonged increases in norepinephrine activity, and altered sensitivity of downstream receptor responses. However, the exact role of noradrenergic transmission remains a mystery.^[2]^

### 1.7. Sleep, insomnia and genetics

To date, there is little understanding of the underlying neurobiological mechanisms of insomnia in the academic community or furthermore little knowledge of any of the complex features of insomnia. As a result, recent studies have taken GWAS studies and none of them so far have addressed the speculation that the risk variables predisposing to the onset of insomnia may be different from those leading to its permanence or chronicity.^[10]^ In contrast, Lind et al^[34]^ conducted a retrospective analysis of candidate gene studies on insomnia and found that insomnia is associated with genetic polymorphisms related to other psychiatric disorders. For example, genes are involved in 5-hydroxytryptamine transport or metabolism.^[35]^ One study found that Apoε4 allele carriers had an increased likelihood of developing insomnia,^[36]^ and overall sleep disturbance measured by applying the Pittsburgh sleep quality index (PSQI) was not significantly associated with dopamine-regulated catecholamine-O-methyltransferase.^[37]^

The GWAS approach is considered more appropriate than the Candidate gene studies approach because complex diseases such as insomnia are highly polygenic, that is, their pathogenesis is determined by any combination of variants among many genes rather than by a specific gene. However, GWAS methods currently face a major dilemma in that they require large samples and a large number of statistical tests. This problem has been solved to some extent with the advent of million-dollar study samples; however, the current need for large cohorts for detailed clinical diagnosis of insomnia remains unresolved.

Recently, a genome-wide analysis involving 1,331,010 replicated MEIS1, MED27, IPO7, and ACBD4, providing strong support for the polygenic nature of insomnia risk.^[36]^ This study identified 956 genes associated with at least 1 of 4 different strategies. The 4 strategies used were locus mapping, eQTL, chromatin mapping, and genome-wide gene-based association analysis. Among these genes, 62 were consistently affected by the 4 different strategies. However, GWAS studies have explained only a small fraction of the phenotypic variation in insomnia, that is, 2.6% of the largest genome-wide association data.^[38]^ Theoretically, if all genetic variants affecting insomnia are known and all their effects can be correctly estimated, the maximum variance explained could be equal to the heritability estimated by meta-analysis (44%).^[39]^ Large differences between the variance explained by GWAS and heritability estimates are common in complex shapes and are referred to as “heritability deficits.” Despite the increasing sample sizes studied and the occasional sensitive and specific phenotypes, if the “heritability deficit” remains large, 1 may see value in GWAS studies of insomnia,^[38]^ as an important value of GWAS is that it reveals clues involving specific biological functional pathways, tissues, and cell types.

### 1.8. Insomnia: Risk factors

Insomnia increases exposure to many risks, including obesity,^[40]^ type 2 diabetes,^[40]^ cardiovascular disease^[41]^ and even an increased risk of suicide.^[42]^ Notably, poor sleep quality is a much stronger predictor of future health problems than short sleep duration.^[43]^ In some individuals, insomnia may persist long after the initial trigger has passed, and some may try to cope with insomnia by adopting bad habits such as drinking alcohol before bed, which may in turn exacerbate the symptoms of insomnia.^[44]^ Moreover, insomnia may trigger PTSD.^[45]^ Indeed, high-quality sleep may prevent poor mood regulation and anxiety in veterans with PTSD.^[46]^

Insomnia has a bidirectional association with psychiatric disorders^[44]^ and gastroesophageal reflux,^[47]^ and among all psychiatric brain disorders, insomnia is probably the most common and burdensome co-morbidity.^[10]^

### 1.9. Polysomnography

Polysomnography (PSG) is a multichannel nocturnal sleep study that is considered the gold standard for semi-objective quantification of sleep. PSG is not strictly required for the diagnosis of insomnia, but we can rule out other possible causes of sleep disruption, such as sleep apnea and periodic limb movements during sleep, by assessing PSG. Contrary to what the name insomnia implies, the EEG of patients with insomnia shows features of sleep in a fragmented manner, manifesting as interrupted wakefulness and sleep transitions, and a meta-analysis showed that the PSG variable reflecting interrupted sleep continuity is the most powerful PSG feature of insomnia.^[48]^ Meta-analysis of PSG in patients with insomnia compared to well-sleeping individuals showed that the greatest effect of the difference between groups of patients with insomnia was a higher number of nocturnal awakenings and, therefore, less efficient sleep. The total sleep time was consequently reduced owing to the reduction in N3 sleep and rapid eye movement sleep. Sleep instability is also manifested by a greater tendency to switch to sleep states at lower depths, which makes it difficult for insomniacs to reach stage N3.^[49]^ In contrast, once stage N3 is reached, sleep in insomnia is more similar to that of normal sleepers, without a significant increase in the probability of switching to a state of lower sleep depth or other indicators of classical instability.^[49]^ However, recent data-driven analysis techniques have revealed that the PSG of insomniacs is characterized by the simultaneous presence of shallow sleep, even in the deepest sleep states.^[13]^

### 1.10. Rooted neurobiology: TCM theory, mind-brain axis and insomnia

#### 1.1.10. Modern Chinese medicine perception of insomnia.

Insomnia is often chronic (>3 months), yet the existing problem is that modern medicines are often recommended for short-term use.^[27]^ Owing to the limitations of medication use, including potential dependence and resistance to long-term use,^[50]^ and the recognition of excessive anxiety factors in the insomnia trajectory, non-pharmacological approaches and traditional Chinese medicine treatments have received increasing attention in the last 2 decades.^[51]^ Cognitive Behavioral Therapy for insomnia has been recommended as the first-line treatment for insomnia, and TCM treatment has both traditional herbal treatment components and non-pharmacological treatments, such as acupuncture and tui-na, emphasizing the combination of pharmacological and non-pharmacological treatments, which are compatible with cognitive behavioral therapy for insomnia in many aspects and can be mutually informed to promote understanding and treatment of insomnia.^[52]^

Based on the theory of TCM, human sleep is inseparable from the tranquility of the “heart and mind,” and when the “heart and mind are at peace,” 1 can sleep peacefully.^[53]^ The “heart” in the basic theory of TCM is different from the heart in modern medicine, and its role in cardiovascular medicine is broader than that of the “heart,” which is understood as the place where the “mind” or “dwelling mind” is hidden. In a narrower sense, it refers to mental awareness, including memory, perception, and thought.^[7]^ The “heart” is a broad concept that encompasses the functions of the heart in the modern medical sense, and the 2 cannot be clearly distinguished or simply considered as unrelated medical concepts.^[54]^ From a systems biology perspective, “heart” and “mind” are not “objects,” but they are related to anatomical organs and belong to multilevel functions.

#### 2.1.10. The heart-brain axis theory for insomnia (Fig. 2).

Modern medicine has demonstrated a close connection between the heart and brain and has gradually developed the heart-brain axis theory, which states that the heart is the pivotal joint connecting the circulatory system and the higher nervous system.^[55]^ Studies have shown that the heart and brain are combined through the autonomic network, which constitutes the central autonomic network and is the supreme center of autonomic nerves.^[56]^ The central autonomic network regulates the vascular tone, pulsation, cardiac output, and myocardial metabolism of the heart after acting through the humors, endocrine, and autonomic nerves, which emanate from the heart, and the blood pumped from the heart supplies the whole body, including the brain, through the aorta.^[57]^ Several experiments have also shown that in addition to autonomic regulatory centers, cardiac afferent neural inputs can affect higher brain centers involved in emotion processing and perception, and changes in afferent and efferent autonomic activity have been found to correlate with changes in heart rhythm patterns, that is, positive emotions lead to increased heart rhythm coherence, whereas negative emotions lead to heart rhythm disturbances.^[58]^ The interdependence between the heart and other organs supported by the TCM theory is also supported when we consider the neuroanatomical connections that exist between the heart and other organs. Clusters of 36 ANS neurons that regulate organs, such as the heart, lungs, gastrointestinal tract, kidneys, and bladder, are located near these organs and communicate with each other, forming a network that facilitates information exchange. For example, neurons that control the heart and respiratory tract communicate with each other.^[59]^

**Figure 2.**
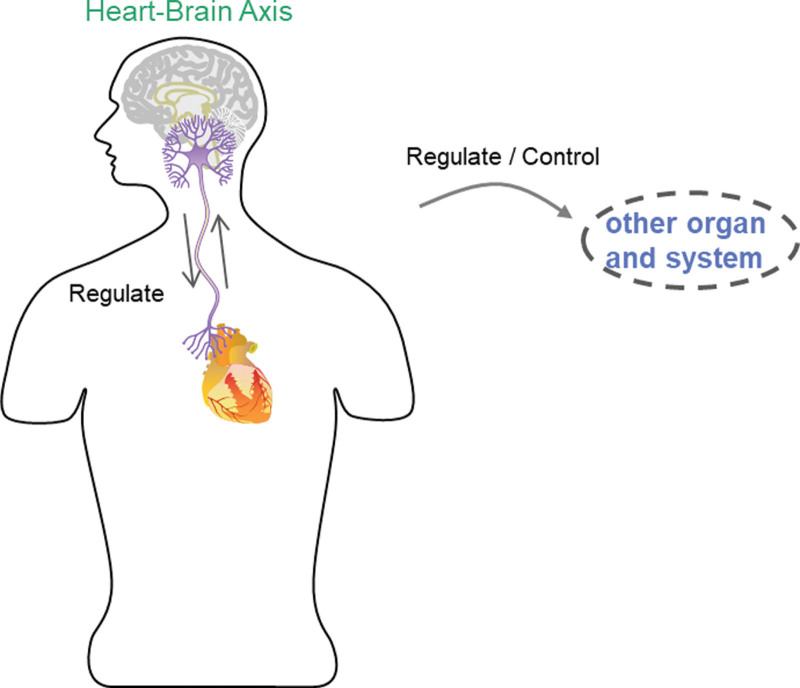
Heart-Brain axis.

Prospective studies have shown that patients with insomnia have an increased likelihood of developing coronary heart disease.^[60]^ Among the many neural connections between the brain and the body’s organ systems, the cardiocerebral axis is an important component that is formed between the brain and the heart through the nervous system,^[61]^ and the vagus nerve is a complex bidirectional system. The vagus nerve originates in the brainstem and projects independently of the spinal cord to many organs in the body cavity, including the heart, respiratory system, and digestive system. Its myelinated branches connect the brainstem to various target organs.^[62]^ These neural pathways allow for direct and rapid communication between brain structures and specific organs. As the vagus nerve contains both afferent and efferent fibers, it facilitates dynamic feedback between brain control centers and target organs to regulate internal environmental homeostasis. Whereas, according to previous studies, cardiac afferents send more signals to the brain than other major organs, approximately 80% to 90%,^[63]^ and brain rhythms exhibit varying degrees of synchronized activity with the heart; for example, brain wave activity and amplitude increase with heart rate. When the heart rhythm is consistent, heart-brain synchronization increases, and these phenomena respond to intercommunication between different biological rhythms.^[64]^ There is evidence that the heart plays a special role in synchronizing activity across multiple systems and levels of organization, through the generation of rhythmic information patterns in the body, the ANS, hormones, stress, and electromagnetic field interactions, as well as other pathways for continuous communication with the brain and body.^[65]^

Studies on vagal activity, mood, and insomnia have found relationships between emotional intensity, vagal function, and sleep in children, with vagal regulation in children assessed by RSA during a baseline and reaction time task,^[66]^ and sleep problems examined by child reports and home monitoring using a wrist activity meter. An increase in emotional intensity predicted a decrease in sleep duration and an increase in nocturnal activity. Poor vagal regulation, characterized by lower inhibition of the reaction time task by the RSA, predicted increased sleep problems. These results suggest that children’s mood and vagal regulation predict unique changes in sleep quality. Another study showed that transcutaneous auricular vagal stimulation ^[67]^ improved sleep quality and prolonged sleep duration in patients with insomnia by reducing functional connectivity within the default mode network, between the default mode network and salience network, and between the default mode network and occipital cortex.

## 2. Results and discussions

### 2.1. Chinese medicine diagnosis and classification for insomnia

TCM prescriptions are based on the theory of TCM, methodological approach of diagnosis and treatment, principles of formulary, and specific use of Chinese medicine in combination to treat insomnia. According to the classification of the Chinese Medicine Clinical Practice Guidelines for Insomnia (WHO/WPO),^[68]^ TCM discriminatory evidence classifies insomnia into liver stagnation and fire, phlegm-heat internal disturbance, yin deficiency and fire, loss of harmony of stomach qi, internal obstruction of blood stasis, heart fire, heart and spleen deficiency, heart and gallbladder qi deficiency, and heart and kidney disconnection. In the process of clinical treatment, the majority of TCM practitioners also use prescriptions and medicines on this basis, and on this basis, they combine modern medicine and other therapeutic methods to study and treat insomnia. Phytosphingosine-1-P may be a potential serum metabolic marker for distinguishing insomnia from liver stagnation and fire from phlegm and fire. Phytosphingosine-1-P may be a typical serum metabolic marker for differentiating insomnia with liver stagnation and fire from phlegm fire and yin deficiency and fire. It is well known that the dialectical typing of TCM is not rigid and unchanging, but will be based on different clinical practices of TCM with regional and contemporary characteristics, and the TCM theory guiding the treatment of insomnia still has dialectical classification methods, such as the 5 Gods typing, which is based on the theory of “5 Sacred Hidden” in the Huangdi Neijing,^[70]^ and is divided into The 5 types of symptoms are: heart does not hide the spirit, liver does not hide the soul, spleen does not hide the will, lung does not hide the spirit, and kidney does not hide the will. Peng Zhipeng^[71]^ showed that the serum dopamine (DA) levels and PSG parameters of the 3 groups differed by analyzing the serum DA levels and PSG parameters in the normal group, indicating that there were differences in the serum DA levels and PSG sleep structure parameters in the 5 divine types of insomnia in TCM; namely, the kidney did not hide the spirit and the spleen did not hide the will. Rui et al^[72]^ similarly found that there were differences in serum MT content and PSQI scores between the liver that does not harbor the soul type and the kidney that does not harbor the will type. Kai-Kai Wang et al^[73]^ designed animal experiments to investigate the differences in 5-HT1A receptors and 5-HT2A receptors in the relevant organs of rats with pulmonary non-hidden soul type and control rats, and showed that the expression of both receptors was significantly elevated, suggesting that 5-HT1A and 5-HT2A receptors are mainly associated with the brain and, to a lesser extent, with the lungs in pulmonary non-hidden soul type insomnia, which may be a characteristic of one of the relevant transmitters.

### 2.2. The practice of TCM prescriptions for insomnia

An important feature of TCM diagnosis and treatment is that different TCM clinicians can prescribe different medications for the same disease; however, this does not mean that the prescription of TCM is completely subjective; there must be some fixed medication patterns in each clinician’s prescription. In a triple-blind, randomized, placebo-controlled, parallel-group clinical trial, each TCM practitioner was found to have 1 or more effective core medication patterns and patients experienced improved sleep quality and longer sleep duration, demonstrating the existence of objective criteria and definite efficacy of TCM prescriptions. It is important to note that although there were only 3 TCM practitioners in the trial, these practitioners were actually considered as treatment rather than a sample, so there was no problem with the small sample size. Hu Kun et al^[75]^ conducted a randomized controlled trial to observe the efficacy of the treatment of insomnia using the classical Chinese medicine formula Sour Date Ren Tang, plus and minus the combination, combined with 5 Elements Music Therapy. The results showed that the sleep treatment was improved and the sleep time was prolonged in the treatment and control groups (*P* < .01, *P* < .05), and the efficacy of the treatment group was better than that of the control group (*P* < .01, *P* < .05). The results showed that it could significantly improve sleep quality and prolong sleep time. The treatment of primary insomnia with a combination of sour date soup and 5-element music was effective in improving sleep quality and prolonging sleep time, which may be closely related to the modulation of neurotransmitters and inflammatory factors by this treatment modality. Shi Jianing et al^[76]^ designed a randomized controlled trial to evaluate the efficacy of the TCM formula Yixin Ningxin Fang on insomnia of the heart and spleen deficiency type, in which patients were randomly divided into 65 cases each in the treatment and control groups. The results showed that Yishen Ningxin Fang could effectively improve the clinical symptoms of patients with heart and spleen deficiency type insomnia, and its mechanism of action was found to be possibly related to the regulation of salivary MT and CORT levels in patients and restoration of the circadian rhythm of salivary MT secretion. Zhang Zhongyang et al^[77]^ designed a randomized controlled trial to investigate the clinical efficacy of Huang Lian Agaricus Tang Plus in treating yin deficiency and fire-exuberance insomnia. This mechanism of action may be related to an increase in the level of 5-HT and a decrease in the level of DA.

### 2.3. Systematic evaluation on TCM prescriptions for insomnia

In terms of systematic evaluation, Hongshi Zhang et al^[78]^ searched electronic databases including PubMed, EmBase, Cochrane library, and China Knowledge Network, and used a random effects model to calculate the total weighted PSQI, Athens insomnia scale, and its 95% confidence interval. Finally, 15 randomized controlled trials including 1500 patients were included in the meta-analysis. and that large-scale, high-quality randomized controlled trials are needed to confirm these results. Xu Fan^[79]^ identified 13 studies involving 1181 participants by Meta-analyzing the efficacy and safety of the Chinese herbal formula Gentian Diarrhea Liver Soup, after searching the databases of PubMed, CBM, CNKI, and VIP, and the results of the analysis showed that the total effectiveness and cure rate were higher than those of the control group, which was statistically significant, adverse effects were lower than those of the Western medicine group, and clinical safety was higher than that of the Western medicine group, which proved the efficacy and safety of Gentian Diarrhea Liver Tang. Hu et al^[80]^ conducted a systematic evaluation of the clinical efficacy and safety of the Chinese herbal formula Zunyao San in the treatment of insomnia with anxiety, and the analysis included 2 randomized controlled trials involving 681 patients, which showed that the combination of prozac with modern medical adjuvant drugs and prozac alone are beneficial in improving sleep quality, prolonging sleep duration, and relieving anxiety in patients with insomnia and anxiety. However, more rigorous and scientific clinical trials are needed for further evaluation due to the relatively low quality and small sample size of randomized controlled trials collected in this systematic evaluation. Proprietary Chinese medicines are Chinese medicinal preparations with objective, standardized, and quantitative index characteristics under the guidance of TCM physical therapy and combined with modern medical technology, and according to a recent meta-analysis,^[81]^ 11 Proprietary Chinese medicines included in the study (Ginseng Astragalus Wu Wei Zi Tablets, Nourishing Blood and Brain Granules, Shu Sleep Capsules, Ginseng Song Yang Xin Capsules, Baile Sleep Capsules, Shu Liver Relief Capsules, Sweet Dream Oral Liquid, Yin Dan Xin Nao Tong The combination of NBZDs (soft capsule) with first-line drugs was effective in the treatment of insomnia. This study made a promising exploration of the systematic study of proprietary Chinese medicines for insomnia.

### 2.4. Acupuncture for insomnia (Fig. 3)

Numerous studies have shown^[82]^ that acupuncture improves sleep quality and prolongs sleep time in patients with insomnia by regulating the activities of sleep factors, such as neurotransmitters, hormones, and cytokines. The specific mechanisms of action are as follows:

**Figure 3.**
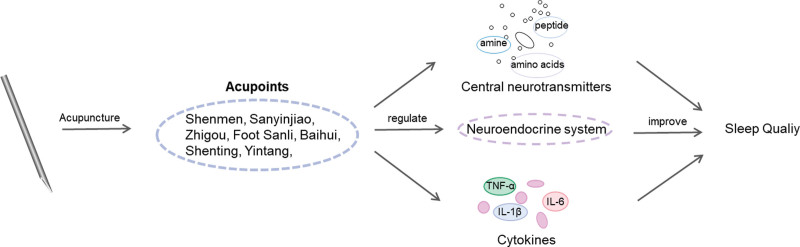
Acupuncture for insomnia.

### 2.5. Effect of acupuncture on central neurotransmitters.

Various central neurotransmitters in the brain have an effect on sleep, and acupuncture affects sleep structure by regulating the levels of 3 types of central neurotransmitters: amines, amino acids, and peptides.^[83,84]^ Li Zhongwen et al^[85]^ designed a randomized controlled trial in which patients with insomnia were acupunctured by acupuncture of Shenmen and Sanyinjiao, and the results showed that acupuncture of sleep-Sanyinjiao could effectively improve sleep quality and upregulate serum γ-aminobutyric acid (GABA) and 5-HT levels in patients. Li Wenkang et al^[86]^ used para-chlorophenylalanine (PCPA) model rats and designed animal experiments according to Experimental Acupuncture; the intervention group was selected to acupuncture bilateral Shenmen, Zhigou, Foot Sanli, and Sanyinjiao points to the inner umbilical ring points, and the results showed that this acupuncture method could improve insomnia symptoms in rats and could upregulate the PCPA model; the results showed that this acupuncture method could improve the symptoms of insomnia in rats, and could upregulate the expression of 5-HT1AR and 5-HT2AR in the hippocampus of the PCPA model.

#### 1.2.5. Acupuncture, cytokines and insomnia.

Cytokines are mostly derived from immune cells and can regulate the immune response and sleep-wake cycle. Acupuncture can regulate sleep mechanisms by modulating immune cytokines such as interleukins.^[87,88]^ Tang Lei et al^[89]^ used the electro-acupuncture method of 5 acupuncture points to randomly divide 40 PCPA model rats into control, model, electro-acupuncture, and diazepam groups, and the results showed that electro-acupuncture of Wuqi Yu could improve the symptoms of insomnia rats by promoting the synthesis of TNF-α and IL-1β in the hypothalamus and alleviating the inhibitory release of 5-HT and its metabolites caused by PCPA. Qian Lala et al^[90]^ designed a randomized controlled trial using Jiaotai Pill combined with acupuncture for Annmian, Xin Yu, Kidney Yu, Shen Men, and Zhao Hai. Sixty patients with insomnia of the heart-kidney disorder were randomized equally into test and control groups. After 4 weeks of treatment, the results showed that Jiaotai Pill combined with acupuncture could better regulate the levels of cytokines TNF-α, IL-6, and IL-1β and improve the sleep quality of patients and treatment of insomnia.

#### 2.2.5. Acupuncture regulates the neuroendocrine system to improve sleep structure.

It is well known that psychological factors affect human sleep and play an important role in the development of insomnia, such as mental tension, anxiety, and depression, which can become stressors and generate neuroendocrine responses, mainly in the sympathetic-adrenomedullary system and the hypothalamic-pituitary-adrenal axis.^[91]^ In contrast, acupuncture can affect sleep architecture, improve sleep quality, and treat insomnia by regulating hormones associated with the sympathetic-adrenomedullary system and the hypothalamic-pituitary-adrenal axis.^[92,93]^ Wu Xuefen et al^[92]^ selected the acupuncture points by meridian and randomly assigned 60 PCPA model rats equally into the blank group, model group, Baihui + Shenmen group, Baihui + Sanyinjiao group, and Baihui + non-meridian non-acupuncture points group, with 12 rats in each group, showing that hypothalamic adrenocorticotropic hormone-releasing hormone, serum adrenocorticotropic hormone, and corticosterone levels were reduced in each acupuncture group. The results showed that the hypothalamic-pituitary-adrenal axis may be one of the mechanisms that affects sleep in rats. Li Jiahuan et al^[94]^ used the method of tuning Shen acupuncture and designed a randomized controlled trial in which 60 patients with insomnia were randomly assigned equally to the treatment group (acupuncture of Baihui, Shenting, Yintang, bilateral Shenmen, bilateral Sanyinjiao) and the control group (acupuncture of bilateral Handsanli, bilateral Fuyun, bilateral Feiyang), and after 4 weeks of treatment, it was found that both groups could improve patients insomnia symptoms, and The PSQI and Fatigue Severity Scale scores of the treated group were significantly lower, with better efficacy in improving insomnia and daytime fatigue, and the plasma melatonin and cortisol levels of patients in the treated group were reduced.

### 2.6. Combining acupuncture and herbal medicine for insomnia (Fig. 4)

In Chinese traditional medicine, both acupuncture and Chinese herbal medicine are effective in the treatment of neuropsychiatric disorders, but Chinese physicians tend to treat insomnia not in a single treatment, but more often in a combined form, most often with acupuncture combined with Chinese herbal medicine. Professor Lin Xianming^[95]^ pointed out that in the treatment of insomnia, “acupuncture should be used to regulate the mind first, and prescription should be used to regulate the body.” The combination of the 2 can treat both the symptoms and root causes, complementing each other.

**Figure 4.**
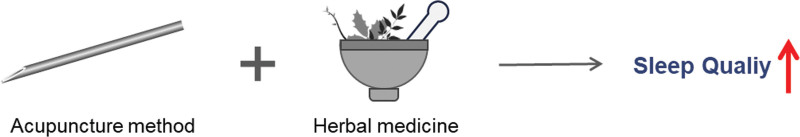
Combining acupuncture and herbal medicine for insomnia.

Xu Kaiquan et al^[96]^ designed a randomized controlled trial using Dong’s Qi acupuncture method (Neiguan, Shenmen, Taixi, Guangyuan, Baihui) combined with Nourishing Heart and Tranquilizing Mind and Dispelling Phlegm Soup (Fu Ling, Han Xia, Xia Gu Cao, Zhu Ru, Chen Pi, Yuan Zhi, Fu Shen, Huang Qi, Yan Hu Suo, Roasted Glycyrrhiza, Sour Jujube and Citrus aurantium) to treat 123 cases of phlegm-damp-internalized insomnia in the combined group, control A group, and control B group. The results showed that all 3 groups could improve the patients’ insomnia symptoms after treatment, and the phlegm-dampness constitution score of the combined group was lower than the remaining 2 groups, the GABA and 5-HT levels were higher than the remaining 2 groups, and the Glu and DA levels were lower than the remaining 2 groups. This indicates that the combined treatment modality can significantly improve the sleep quality of patients with phlegm-damp internal insomnia, improve the phlegm-damp constitution, and regulate and adjust serum GABA and Glu levels. Qin Meiying et al^[97]^ collected 164 patients with insomnia of the heart and spleen deficiency type and randomly and equally assigned them into treatment and control groups. The treatment group received sour jujube soup combined with Ziwu Liujiao acupuncture method, while the control group was administered estradiol tablets. The BDNF and GDNF levels in the treatment group were higher than those in the control group, indicating that this combined treatment modality improved the sleep quality of patients by regulating the immune inflammatory state, inhibiting the inflammatory response, and regulating neurocytokine levels. Ma et al collected 108 patients with insomnia of the heart and spleen deficiency type and divided them into a control group and a treatment group; the treatment group used the combined treatment and the control group used only the tranquilizing acupuncture method. Patients’ sleep quality and PSQI and TCM symptom scores were lower in the treatment group, IL-6 and DA were lower in the treatment group than in the control group, and 5-HT and GABA were higher than in the control group, indicating that the efficacy of the treatment group was better than that of the control group. This combined treatment method was effective in improving patients sleep quality and treating insomnia. Yan Xueli et al^[98]^ used acupuncture (acupuncture of Si Shen Cong, an Mian, Shen Men, San Yin Jiao, Liver Yu, Lung Yu, Feng Chi, and Foot San Li) combined with Xiang Shu Tang (Xiang Shu, Chuan Xiong, Chai Hu, Qing Pi, Sour Jujube, and Hefei Pi) to treat 120 patients with perimenopausal insomnia of liver depression and qi stagnation, randomly and equally assigned into treatment and control groups. The treatment group received the combined treatment, and the control group was given eszopiclone tablets, and the treatment course was 16 weeks. The results showed that insomnia symptoms improved in both groups, and the TCM symptom, PSQI, and Hamilton Anxiety Inventory scores were lower in the treatment group than in the control group. Serum luteinizing hormone and follicle hormone levels decreased significantly, and estradiol levels increased in the treatment group, and the changes were more significant than those in the control group. The significant decrease in serum luteinizing hormone and follicle hormone levels and the significant increase in estradiol levels after combination treatment may be one of the mechanisms for the improvement of sleep-wake regulation by this combination treatment.

## 3. Conclusion

### 3.1. Intersection of TCM and modern medicine

Although it is well known that there are different views of TCM theory in relation to the pathophysiology and neurobiology of insomnia, especially the centrality of “heart” in TCM physiotherapy, some TCM practitioners have started to recognize the fact that “brain” is the highest center of the whole body after the Ming and Qing dynasties. However, given the multi-centered and multi-level theoretical characteristics of Chinese medicine, which are not based on systematic anatomy alone, the “heart” is still regarded as the “sovereign’s official” in the education and medical practice of Chinese medicine. Therefore, in the education and medical practice of Chinese medicine, the heart is still regarded as sovereign. Modern medicine, on the other hand, from anatomy and biomedicine, has always firmly regarded the function of the heart in Chinese medicine as a function of the brain. However, theories have not remained unchanged, and the development of the heart-brain axis theory and the study of brain-heart function interactions have provided meaningful insights for cardiology and neuroscience. With respect to biological signal processing, this interaction primarily involves the linear dynamics of the nerve and heartbeat, expressed through time and frequency-domain correlated features. However, the dynamics of the central and autonomic nervous systems exhibit nonlinear and multifractal behavior, and the extent to which this behavior affects brain-heart interactions is currently unknown. According to a recent study, Catrambone et al^[99]^ reported a new signal processing framework designed to quantify nonlinear functional brain-heart interactions in non-Gaussian and multi fractal domains, which relies on a maximum information coefficient analysis between nonlinear multi-scale features obtained from the EEG spectrum and an inhomogeneous point process model of heartbeat dynamics. The conclusions show that significant physical and sympathetic changes, such as those induced by cold-pressure stimulation, have an impact on brain-heart function interactions beyond second-order statistics, thus extending to multi fractal dynamics. These results provide a platform for identifying novel neurologically targeted biomarkers. These studies increasingly suggest that both the heart and the brain may not be central to the body and that there may be a possibility of a binary “heart-brain” central framework.

### 3.2. Chinese medicine treatment of insomnia: challenges and shortcomings

The efficacy of evidence-based TCM treatment is inevitably limited by the professionalism and ability of TCM practitioners, which indicates that TCM treatment for insomnia is currently unable to achieve objective, standardized, and quantified treatment effects, which in turn will limit the progress of TCM research. At present, research on insomnia in Chinese medicine is not objective, standardized, and quantified because of the diversity and variability of evidence-based typing and the fact that Chinese medicine formulas are based on evidence-based treatment and have the characteristics of “adding and subtracting at the moment and changing randomly” in clinical practice. This has led to the fact that research on the identification model of TCM, which is the core of treatment, has not been widely recognized internationally, and the value of the research has been seriously affected. The value of acupuncture in the treatment of insomnia has become increasingly widely recognized because some studies are based on modern medical research and because acupuncture points can quantify indicators and results to a certain extent, but it also has its own shortcomings. Randomized controlled trials of acupuncture and animal experiments are limited by the research method itself, and it is difficult to achieve a large sample of observations, which is often in the thousands. However, it is difficult to observe large samples of thousands of people.

In the process of research, TCM researchers also face differences in their understanding of modern medicine, which suggests that our research still has a long way to go.

### 3.3. Future perspectives

Despite the challenges faced, this has not dampened the enthusiasm of TCM researchers. Cheng-Yong Liu et al^[51]^ designed a randomized controlled protocol of acupuncture combined with cognitive behavioral therapy for the treatment of insomnia. It is of high scientific value to explore the possibility of combining these 2 treatments. At the same time, Jin Yarong et al^[100]^ used the combination of tonic kidney and Anzhi formula with cognitive behavioral therapy to treat insomnia and designed a randomized controlled trial, which showed that the efficacy of the combined treatment was better than that of cognitive behavioral therapy alone. These studies have made a promising exploration of TCM for the treatment of insomnia.

We must understand that TCM is rooted in TCM tradition; we must focus on the value of TCM classics, and should actively explore these classics, Huang Di Nei Jing, Theory of Typhoid, and Qian Jin Yao Fang, and use modern science and technology to verify the value of these classics, to study and promote the development of TCM theory, and the progress of TCM clinical practice.

## Author contributions

**Conceptualization:** Jie Wang.

**Data curation:** Jie Wang, Haishen Zhao, Kejun Shi, Manya Wang.

**Formal analysis:** Jie Wang.

**Funding acquisition:** Jie Wang.

**Investigation:** Jie Wang.

**Methodology:** Jie Wang.

**Project administration:** Jie Wang.

**Resources:** Jie Wang.

**Software:** Jie Wang.

**Supervision:** Jie Wang.

**Validation:** Jie Wang.

**Visualization:** Jie Wang.

**Writing – review & editing:** Jie Wang.
